# The Role of Gasotransmitters in Gut Peptide Actions

**DOI:** 10.3389/fphar.2021.720703

**Published:** 2021-07-20

**Authors:** Wout Verbeure, Harry van Goor, Hideki Mori, André P. van Beek, Jan Tack, Peter R. van Dijk

**Affiliations:** ^1^ Translational Research Center for Gastrointestinal Disorders, KU Leuven, Leuven, Belgium; ^2^ Departement of Endocrinology, University Medical Center Groningen, Groningen, Netherlands

**Keywords:** gut peptide, nitric oxide, carbon monoxide, hydrogen sulfide, gasotransmitters

## Abstract

Although gasotransmitters nitric oxide (NO), carbon monoxide (CO) and hydrogen sulfide (H_2_S) receive a bad connotation; in low concentrations these play a major governing role in local and systemic blood flow, stomach acid release, smooth muscles relaxations, anti-inflammatory behavior, protective effect and more. Many of these physiological processes are upstream regulated by gut peptides, for instance gastrin, cholecystokinin, secretin, motilin, ghrelin, glucagon-like peptide 1 and 2. The relationship between gasotransmitters and gut hormones is poorly understood. In this review, we discuss the role of NO, CO and H_2_S on gut peptide release and functioning, and whether manipulation by gasotransmitter substrates or specific blockers leads to physiological alterations.

## Introduction

A variety of gastrointestinal hormones provide the regulation of digestive processes. Aberrant gut peptide release or function has been implicated in disorders of the gastrointestinal tract and may lead to several symptoms as shown in dumping syndrome, irritable bowel syndrome, functional dyspepsia and gastroparesis (Camilleri, 2014; Van den Houte et al., 2020). Enteroendocrine cells in the stomach, small intestine and pancreas receive neuronal and nutritional information and accordingly secrete or contain their content. Emerging evidence shows a potential role of gasotransmitters in the regulation and function of gut hormones. Small gaseous signaling molecules, such as nitric oxide (NO), carbon monoxide (CO) and hydrogen sulfide (H_2_S), can freely pass the cell membrane and manage functions in these enteroendocrine cells (Wang, 2002).

**FIGURE 1:**
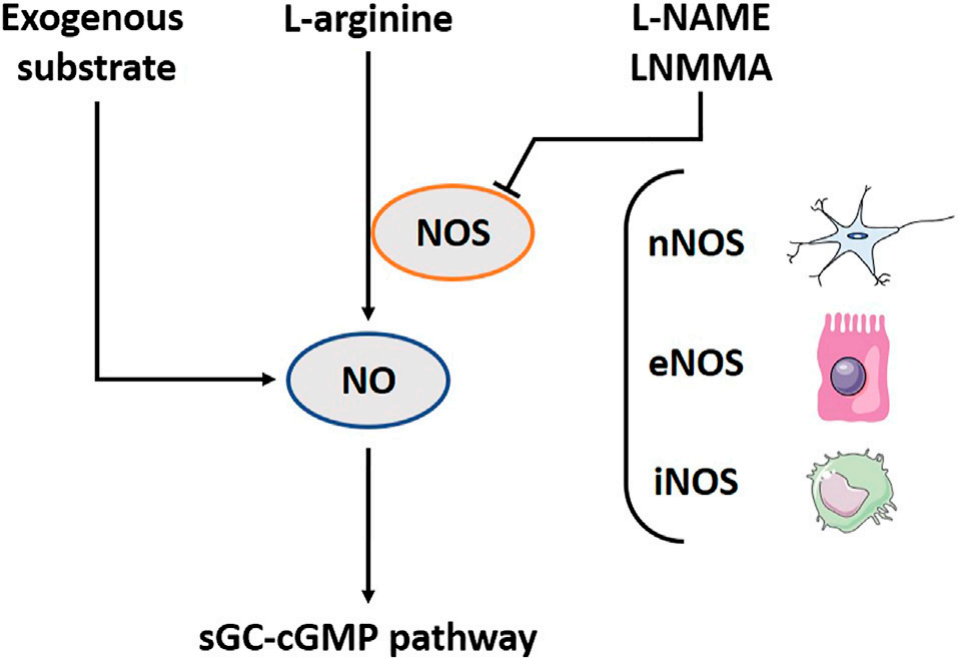
The nitric oxide (NO) pathway including involved substrates, enzymes and downstream pathway. l-arginine amino acid is the substrate from which NO is generated by the enzyme NOS. Three different NOS-isoforms exist: neuronal NOS (nNOS), endothelial NOS (eNOS) and inducible (inflammatory) NOS (iNOS). In addition, several exogenous substrates exist to induce the NO-downstream pathway. l-NAME and LNMMA block NOS function. The biological functions of NO are related to the activation of sGC; subsequently influencing cGMP. NOS, Nitric oxide synthase; l-NAME, N(ω)-nitro-l-arginine methyl ester; LNMMA, L-N(G)-monomethyl arginine; sGC, Soluble guanylyl cyclase; cGMP, Cyclic guanosine monophosphate.

NO, the first discovered gasotransmitter, has been extensively studied. It is a key regulator in processes such as blood vessel dilatation (Vallance et al., 1989). It is also involved in muscle relaxation such as the relaxation of the fundic area after a meal, called the gastric accommodation (Tack et al., 2002). Arginine amino acid is the substrate from which NO is generated by the enzyme nitric oxide synthase (NOS) (Figure 1). Three different NOS isoforms exist: neuronal NOS (nNOS), endothelial NOS (eNOS) and inducible (inflammatory) NOS (iNOS). The first two produce the required concentrations in healthy situations to regulate physiological functions as earlier mentioned. The latter is mainly present in macrophages and is able to produce high amounts of NO to cope with a stress factor as e.g., an infection (Förstermann and Kleinert, 1995). Exogenous NO precursors can be found as food supplements (Bescós et al., 2012), or synthetic forms with longer activity like isosorbide mononitrate (Abshagen, 1992). The nitroaspirin (NCX-4016) is a NO releasing derivative of aspirin that inhibits platelet aggregation induced by adenosine diphosphate and thrombin agonists as an action of aspirin, and protects the stomach from aspirin-induced gastric mucosal damage by NO (Burgaud et al., 2002; Fiorucci et al., 2003). Inhalation of low NO concentrations (INOmax) leads to bronchodilation (Bin-Nun and Schreiber, 2008). Sildenafil does not contain a substrate, but it interacts with the downstream NO-pathway as a phosphodiesterase five inhibitor (Corinaldesi et al., 2016). l-arginine analogues, like N(ω)-nitro-l-arginine methyl ester (l-NAME) and L-N(G)-monomethyl arginine (LNMMA), are chemical compounds, which block NOS function, a required tool for further research (Robbins and Grisham, 1997; Kopincová et al., 2012). Furthermore, NO is extremely reactive and has a half-life time of mere seconds (Hakim et al., 1996). It is supposed to be mostly active at the site of production and the surrounding cells.

**FIGURE 2:**
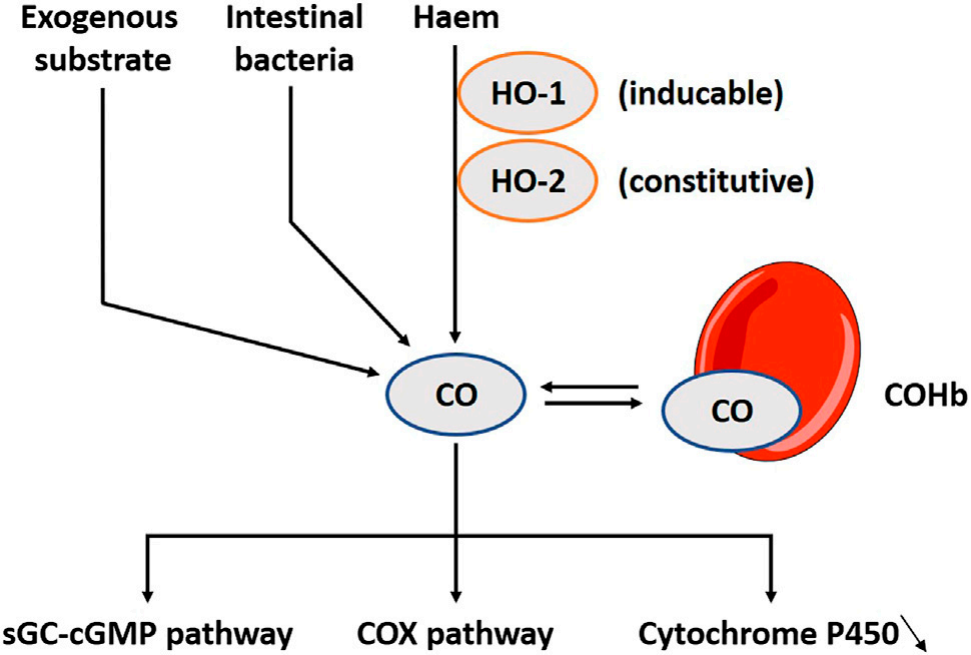
The carbon monoxide (CO) pathway including involved substrates, enzymes and downstream pathways. CO is produced during the catabolism of haem by two oxygenase proteins, HO-1 and HO-2, but it can also be released in small amounts by intestinal bacteria. Interestingly, several exogenous substrates to induce the pathway exist. CO prefers binding hemoglobin, and this complex has a long half-life time up to 4 h. CO’s biological functions are related to the activation of sGC; subsequently influencing cGMP, but other pathways such as the COX or inhibition of cytochrome P450 have also been explored. HO-1, Haem oxygenase 1; HO-2, Haem oxygenase 2; COHb, CO-haemoglobin complex; sGC, Soluble guanylyl cyclase; cGMP, Cyclic guanosine monophosphate; COX, Cyclo-oxygenase.

CO has colloquially received a bad connotation. However, scientists now start to understand its physiological relevance (Kim et al., 2006). Haem is catabolized by two oxygenase proteins: haem oxygenase 1 (HO-1) which is additionally activated during inflammation or a period of oxidative stress, and haem oxygenase 2 (HO-2) which is constitutively active (Maines, 1997; Motterlini et al., 1998) (Figure 2). HO-1 can be found in human gastric epithelial cells and in inflammatory cells in the lamina propria. It has been suggested that nutritional modulations may be used as an intervention for local inflammation (Coëffier et al., 2002). HO-2 is more broadly expressed than HO-1 and can be found in the brain, liver and vascular endothelial cells (Maines, 1988; Ewing and Maines, 1992). In the gut, it is present in the myenteric and submucosal plexus in the jejunum (Matsuda et al., 2010). In Hirschsprung’s disease, the impaired inhibitory motor control is a prominent symptom, and associates with downregulation in HO-2 mRNA (Chen et al., 2002). Under aerobe conditions, luminal bacteria also produce CO (Engel et al., 1972), though this is considered a small proportion of the total production (Hermann et al., 2012). This gasotransmitter differentiates itself from NO and H_2_S as a more stable molecule. It prefers binding hemoglobin, and this complex has a long half-life time up to 4 h (Pan et al., 2020). Its biological functions are related to the activation of soluble guanylyl cyclase (sGC); subsequently influencing cGMP, but other pathways such as the cyclo-oxygenase (COX) or inhibition of cytochrome P450 are also reported (Mancuso et al., 1997). CO is not as effective for activating sGC as NO (Stone and Marletta, 1994), but when intracellular levels of NO are low, CO functions as a back-up system (Wu and Wang, 2005; Moustafa and Habara, 2014a)*.* Exogenous inhalation of CO gas induces a wide range of beneficial responses (Foresti et al., 2008). CO releasing molecules (CORMs) are metal carbonyl complexes releasing CO dose-controlled and tissue-specific. These potential clinical applications are dominantly tested in cell-lines and animal models with little to none studies done in humans (Naito et al., 2016; Steiger et al., 2016). A broad range of CORMs functions are already discussed in depth elsewhere (Foresti et al., 2008; Ismailova et al., 2018; Ling et al., 2018).

**FIGURE 3:**
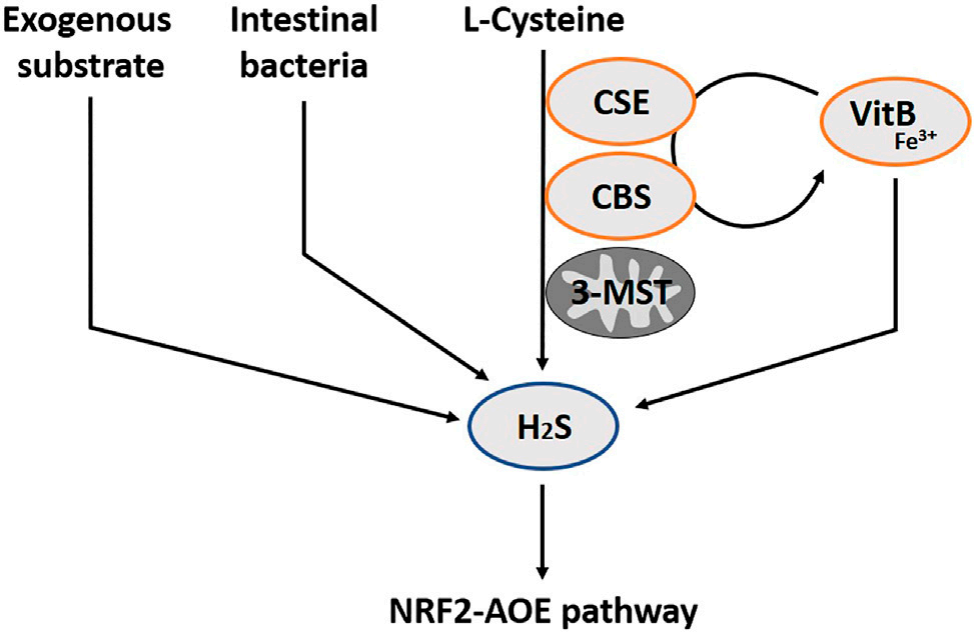
The hydrogen sulfide pathway (H_2_S) including involved substrates and enzymes. H_2_S is produced through enzymatic pathways of CSE, CBS and 3-MST. In addition, intestinal bacteria can also secrete H_2_S, and several exogenous substrates exist. Vitamin B6 and iron are also required for the non-enzymatic synthesis of H_2_S in blood. H_2_S exerts antioxidant effects through several mechanisms, such as increasing expression of AOE, by activating the transcription factor NRF2. CSE, Cystathionine gamma-lyase; CBS, Cystathionine beta-synthase; 3-MST, 3-mercatopyruvate; VitB, Vitamin B6; AOE, Antioxidant enzymes; NRF2, Nuclear factor erythroid-derived 2-like 2

H_2_S has only recently joined the group of gasotransmitters. Therefore, its relevance has been less well studied. There are two enzymatic pathways in the production of H_2_S, cystathionine gamma-lyase (CSE) and cystathionine beta-synthase (CBS), the so-called transsulfuration pathways (Wang, 2002) (Figure 3). A third enzymatic pathway primarily exists in mitochondria, the cysteine catabolic pathways by 3-mercatopyruvate sulfurtransferase (3-MST) (Shibuya et al., 2009). The pyridoxal phosphate cofactor form of vitamin B6, in the presence of Fe^3+^, is essential to catalyze the enzymatic H_2_S production via CSE and CBS. In addition, vitamin B6 and iron are also required for the non-enzymatic synthesis of H_2_S in blood (Yang et al., 2019). It is no surprise that a vitamin B6 deficiency translates in reduced H_2_S synthesis (Hughes et al., 2017). CBS is mainly expressed in brain neurons, neurons of the enteric gut nerve system and smooth muscle (Kimura, 2014). CSE is more prominent in the smooth muscles of the vascular system, and is wide spread in other smooth muscle tissues (e.g. of the gut). There is also expression in the brain, albeit that CBS is represented as the dominant enzyme at this site (You et al., 2011). Similar to NO and CO, H_2_S is involved in gastro-intestinal (patho-)physiology, including pro- and anti-inflammatory activities, control of motility and vascular tone (Cipriani and Mencarelli, 2011; Medani et al., 2011; Chan and Wallace, 2013; Linden, 2014). Intestinal bacteria are an additional source of H_2_S in the gut lumen, which might push H_2_S concentrations locally above the healthy limit, and evokes disease in some cases (Blachier et al., 2019). Allicin is a natural H_2_S donor simply found in vegetables like garlic and onions (Benavides et al., 2007). Sulfide salt pills are also used, with a preference for CaS instead of NaSH due to its stability (Li et al., 2009; Wen et al., 2018). There is also a synthetic slow-releasing donor, GYY4137, but this is not yet in the phase of application in humans (Powell et al., 2018; Wen et al., 2018).

In the next section, we summarize the state of knowledge on the interaction between gasotransmitters and the gastrointestinal hormones: gastrin, cholecystokinin (CCK), secretin, motilin, ghrelin, glucagon-like peptide (GLP) -1 and -2.

## Gastrin

**FIGURE 4:**
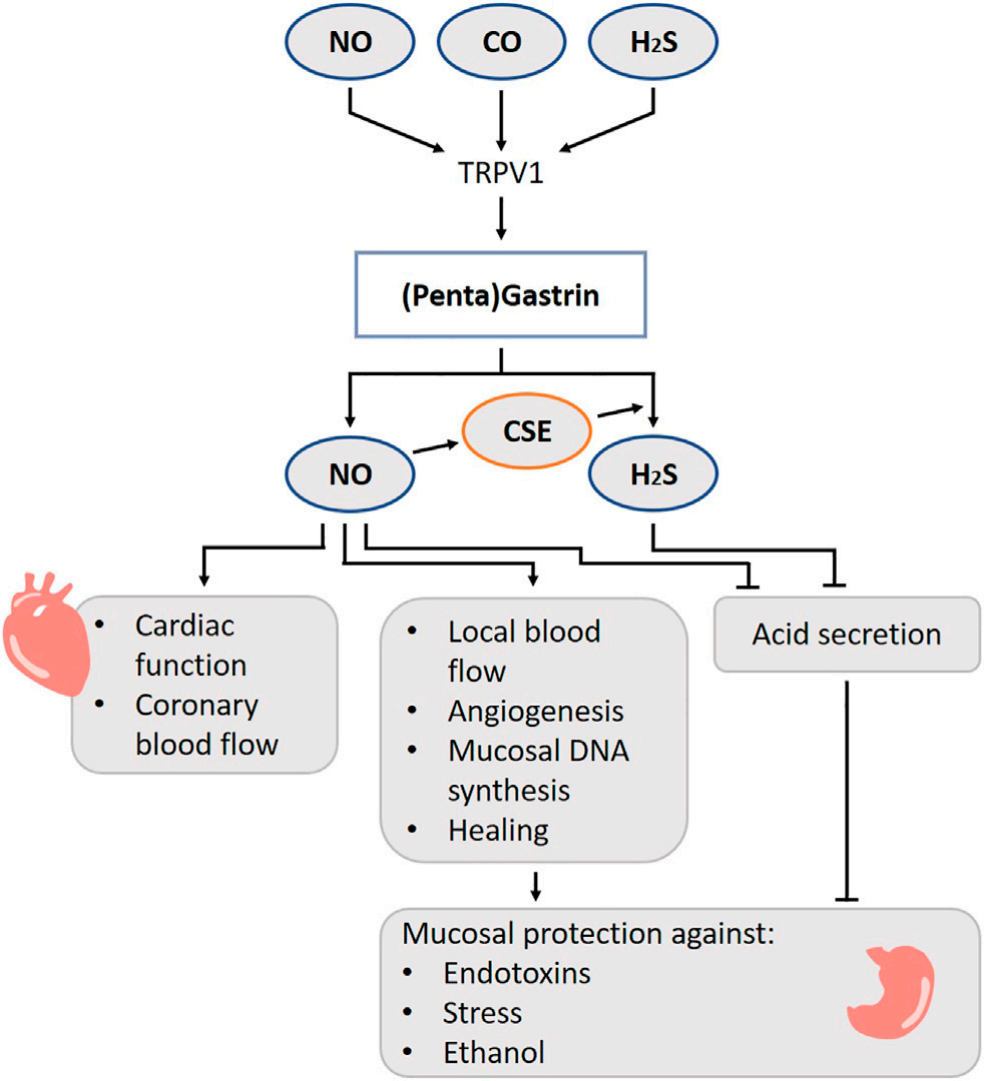
Role of gasotransmitters on gastrin release and its physiological functions. Solid lines are clear evidence–based on blockers and substrate administration. Gaseous molecules may regulate gastrin release via TRPV1. There is a beneficial relation between NO and gastrin leading to hyperemia, increased local blood flow, angiogenesis, mucosal DNA synthesis and increased healing. NO and H_2_S governs the gastric acid output. Taken all together, gastrin performs a stomach protecting function via gasotransmitters. NO, Nitric oxide; CO, Carbon monoxide; H_2_S, Hydrogen sulfide; TRPV1, Transient receptor potential vanilloid subtype 1; CSE, Cystathionine gamma-lyase.

Gastrin is a peptide hormone released from the G-cells situated mainly in the antrum and less dispersed in the non-antral stomach, small intestine and pancreas (Rehfeld, 1998). The presence of food stimulates the G-cells in different ways: directly by sensing proteins and its digested forms (Rehfeld, 1998), by the pyloric antrum (Sugawara et al., 1970), by activation of the vagal nerve and by responding to changes in luminal pH (Chu et al., 1999). Capsaicin, which causes a “burning sensation” and which is present in e.g. chili peppers, stimulates gastrin secretion from isolated human antral glands (Ericson et al., 2009). Capsaicin-sensitive afferent neurons also participate in the secretion of gastrin by luminal alkalization or acidification in a rat model (Nojima et al., 2000). The capsaicin receptor, the transient receptor potential cation channel subfamily V member 1 (TRPV1), functions as a detector for other chemical and physical stimuli such as a temperature rise, pH change, mechanical stimulation, osmotic pressure change and gaseous molecules such as NO, H_2_S and CO (Takahashi et al., 2012) (Figure 4). Thus, gaseous molecules may regulate gastrin release via TRPV1.

Gastrin stimulates parietal cells to promote acid release during the gastric phase of digestion. It acts both directly on the parietal cell and indirectly via binding onto cholecystokinin 2(CCK2)/gastrin receptors on enterochromaffin-like cells in the stomach, which in turn release histamine (Rehfeld, 1998). In addition, gastrin is known to cause chief cells to secrete pepsinogen (Fiorucci and McArthur, 1990), to increase antral muscle contractions (Cameron et al., 1970), to strengthen antral contractions against the pylorus, and to relax the pyloric sphincter which increases the rate of gastric emptying (Fisher et al., 1973), to induce relaxation of the ileocecal valve (Vadokas et al., 1997), to stimulate pancreatic secretions and gallbladder emptying (Valenzuela et al., 1976), to stimulate cell proliferation in gastrointestinal mucosa (Watson et al., 2006), to play a role in protective activity against ethanol damage of gastric mucosa (Konturek et al., 1995a; Konturek et al., 1995b) and to increase lower esophageal sphincter (LES) tone (Castell, 1978). When these physiological processes do not follow the normal course of events, understanding the governing role of gasotransmitters on these pathways might help to restore the divergence.

One study reported that endogenous NO did not directly modulate acid secretion induced by pentagastrin, a gastrin agonist, in anesthetized rats (Pique et al., 1992). However, there is a body of evidence that favors an inhibitory role of NO on gastric acid secretion in isolated human gastric glands and in an *in vivo* rat and dog model (Bilski et al., 1994; Stroff et al., 1994; Berg et al., 2004). In case of a disease, there is often an inhibition of gastric acid secretion, possibly intended as survival mechanism to protect against further degradation of the stomach wall. This phenomenon has been attributed to the presence of endotoxin. In rats, administration of *E. Coli* endotoxin abolishes the acid secretion induced by pentagastrin. However, after pre-treatment of these rats with both l-NAME and a platelet-activating factor receptor antagonist, the gastric acid output is restored. This suggests that formation of NO may be involved in endotoxin-induced inhibition of acid production (Martinez-Cuesta et al., 1992). Besides for endotoxins, a similar response is observed after administration of oxytocin, elevation of body temperature or reduction in blood pressure as proxies for somatic stress in rats (Esplugues et al., 1996). Administration of l-NAME in the dorsal motor nucleus of the vagus nerve reversed the acid-inhibitory effects of aforementioned stimuli. The *in vivo* action of NO in the rat brainstem in modulation of gastric acid has also been reported by others (Beltrán et al., 1999; García-Zaragozá et al., 2000; Quintana et al., 2001) and seems to be calcium dependent and specifically related to nNOS (Barrachina et al., 1995a; Barrachina et al., 1995b; Esplugues et al., 1996; Uribe et al., 1999).

HO-1 and -2 are expressed in the mucosa of the rat and monkey fundic glands in cells containing the H^+^K^+^-ATPase, markers for parietal cells (Hu et al., 1998; Ueda et al., 2009; Takagi et al., 2016). HO-2 immunopositive cells are also present in the pyloric part of the stomach, although to a lesser extent. The majority of these cells also seem to express gastrin, but not somatostatin (SST) or serotonin (Hu et al., 1998). Whether CO production has a physiological relevance for parietal cells and gastrin cells is not known to date. More studies are required looking into the effect of CO on gastrin, histamine and acid release, e.g., using HO-blockers.

H_2_S governs the gastric acid output to avoid development of ulcers. It contains gastric acid overproduction, releases bicarbonate in the duodenum and stimulates repair after an injury in rat *in*- and *ex vivo* models (Wallace, 2012; Mard et al., 2014; Takeuchi et al., 2015). Both CBS and CSE are present in the mucosa of the rat stomach, although only CSE expression is targeted by anti-inflammatory nonsteroidal drugs (Fiorucci et al., 2005). Mard S. and colleagues showed that gastric acid secretion, evoked by pentagastrin or gastric distention, upregulates CSE, and not CBS, expression in rats. NO is an essential step for the translation of CSE mRNA to the enzyme, and thus eventually for H_2_S production (Mard et al., 2015).

When the need for acid release decreases, other gut peptides are released: SST (Bloom et al., 1974), secretin (Itoh et al., 1975), glucose-dependent insulinotropic polypeptide (GIP), vasoactive intestinal peptide (VIP) (Villar et al., 1976), and calcitonin (Hornum et al., 1976); and all of these exert inhibitory effects on the G-cells.

There is a beneficial interaction between gastrin and NO with respect to preserving mucosal integrity in response to toxins. The protective effects of gastrin on ethanol-induced mucosal damage are eliminated when NO synthesis is blocked and restored when l-arginine is administered in rat models *in vivo* (Stroff et al., 1994; Konturek et al., 1995a; Konturek et al., 1995b). In the context of gastric ulcers, there is also a beneficial relation between administration of NO and gastrin concentrations leading to hyperemia, increased local blood flow, angiogenesis, mucosal DNA synthesis and, ultimately, increased healing in rats (Brzozowski et al., 1995; Tatemichi et al., 2003).

In historical experiments, administration of the CCK2R (gastrin receptor) agonist pentagastrin not only increased gastric blood flow, but also affected arterial blood pressure and myocardial contractility through NO and the parasympathetic system in anesthetized rats and cats (Pawlik et al., 1987; Walder et al., 1990). Stimulation of the CCK1/2R by gastrin-17, one of the two major postprandially released forms of gastrin, stimulates endothelial NO production in porcine coronary arteries (Grossini et al., 2012) and results in a dose-related increase in cardiac function and coronary blood flow (Grossini et al., 1985; Grossini et al., 2011). It is noteworthy, that besides stimulation of CCK1/2R, β2-adrenoreceptors-related NO release is likely to be involved in the effects of gastrin on the porcine coronary endothelial cells *in vivo* (Grossini et al., 2012). Based on these results, we suggest that gastrin may be involved in the increase in cardiac output associated with digestion.

Concerning the effects of NO and gastrin on motility, a small randomized trial showed that the NO-donor molsidomin decreased LES pressure. However, this is not associated with changes in plasma concentrations of gastrin (Brzozowski et al., 1995). In addition, NO does not seem to be involved in the gastrin-induced relaxation of the canine stomach (Schuurkes and Meulemans, 1994; Meulemans et al., 1997).

The HO-2 enzyme is expressed in neurons of the myenteric plexus and submucosal plexus from stomach and jejunum in humans, where the expression pattern with NOS overlaps for 40% (Miller et al., 2001). CO induces the production of cGMP which may increase cAMP levels by inhibiting phosphodiesterases, eventually leading to a hyperpolarization which relaxes smooth muscles (Wu and Wang, 2005). Normally, NO downregulates the HO-2 expression (Ding et al., 1999), but in case of O_2_-shortage, the negative feedback on CO production is no longer present and CO takes over several governing functions (Wu and Wang, 2005). CO is responsible for the nonadrenergic/noncholinergic (NANC) relaxations, although the role of gastrin in this pathway is not known (Watkins et al., 2004).

In rodents, H_2_S relaxes gastrointestinal muscles (Gallego et al., 2008), such as the pyloric sphincter (Medeiros et al., 2012). Moreover, administration of l-cysteine and NaHS enhances gastric emptying of a liquid meal. Treatment with a CSE antagonist, dl-propargylglycine, blocks the accelerated gastric emptying. In addition, a TRPV1 antagonist also abolishes the effect, implicating that these channels are involved in the induced relaxation of the pylorus muscles. The l-cysteine and NaHS administration cause no changes in acidity in comparison to the placebo, suggesting that gastrin is probably not involved in this effect (Medeiros et al., 2012).

## Cholecystokinin

CCK is released by enteroendocrine I-cells, located in the duodenal and jejunal mucosa, by luminal exposure to food (French et al., 1995; Geraedts et al., 2010). Fatty acids or their monoglycerides containing twelve or more carbon atoms are the most potent stimuli for CCK release (McLaughlin et al., 1999). Its most important role is in the digestion of fat particles and/or proteins that normally take a long time to be chemically broken down. This is achieved by CCK-induced contraction of the gallbladder, secretion of pancreatic enzymes and relaxation of the sphincter of Oddi (Geenen et al., 1980; Masclee et al., 1989; Schmidt et al., 1991). Furthermore, CCK directly stimulates the suppression of appetite in the brain and inhibits gastric motility and emptying, thereby increasing the time for complete digestion (Chandra and Liddle, 2007). The actions of CCK are mediated by two receptors, designated as CCK1 receptor (CCK1R) and CCK2 receptor (CCK2R). In humans, CCK1Rs are mainly located peripherally such as in the stomach, the intestine, colon, gallbladder muscularis and to a lesser extent in the pancreas (Reubi et al., 1997; Schmitz et al., 2001). CCK1Rs are also expressed in discrete areas in the mouse brain, but in these regions, CCK2Rs dominate (Vialou et al., 2014). In addition, the latter can also be found in the stomach (such as in acid secreting cells) and the pancreas in humans (Nishimori et al., 1999; Schmitz et al., 2001). CCK2 brain receptors exert a complex regulation of dopamine activity in the brain, and thus their involvement in human anxiety states is well documented. Patients with panic disorder are hypersensitive to CCK2R stimulation compared to healthy volunteers (Bradwejn and Koszycki, 2001). CCK stimulates SST release by binding the CCK1Rs on gastric D-cells, thereby indirectly inhibiting gastric acid secretion (Schmitz et al., 2001). CCK receptors are also expressed in neural tissues (Dufresne et al., 2006). Here, CCK acts as a neuromodulator and/or -transmitter and influences processes such as satiety, nociception, and anxiety (Crawley and Corwin, 1994). CCK2R is also known as the gastrin receptor, and this is because gastrin and CCK share their carboxyl-terminal heptapeptide amide, an important region for their biological activity (Miller and Gao, 2008).

**FIGURE 5:**
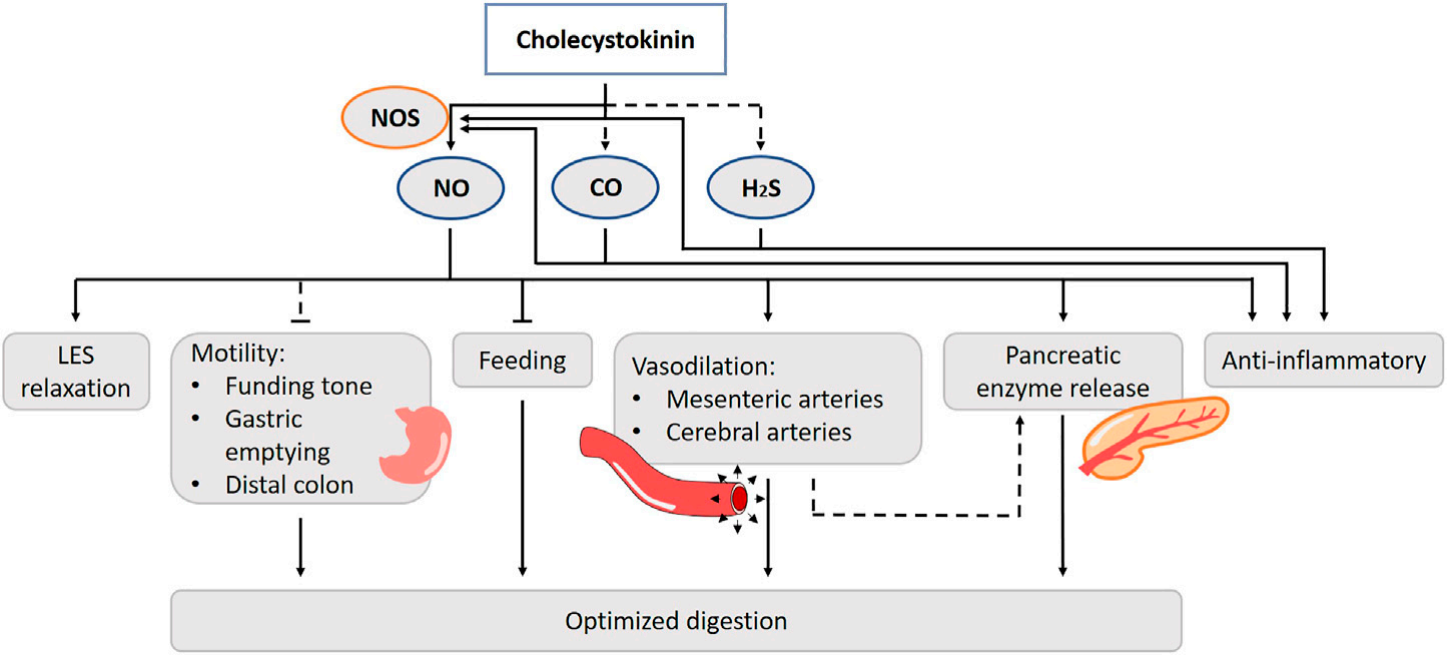
Role of gasotransmitters on cholecystokinin’s (CCK) physiological functions. Solid lines are clear evidence–based on blockers and substrate administration. Dotted lines represent first evidence for the presence of the pathway. CCK stimulates nNOS activity and thus NO release. NO is involved in the cholecystokinin effect on LES relaxation, intestinal vasodilation and food intake regulation. The CCK-NO-pathway of intestinal motility has only been hypothesized in literature. CCK anti-inflammatory actions are evoked by reducing vascular and macrophage iNOS-derived NO production. The involvement of CO and H_2_S in these functions have only been suggested, but lack experimental prove. NO, Nitric oxide; CO, Carbon monoxide; H_2_S, Hydrogen sulfide; NOS, Nitric oxide synthase; LES, Lower esophageal sphincter.

Among various physiological actions of CCK, NO has been shown to be involved in intestinal motility, intestinal vasodilation and food intake regulation. Cholecystokinin-8 (CCK8) administration increases the occurrence of transient LES relaxations through the activation of CCK1Rs in anesthetized dogs. Pretreatment with the NOS inhibitor abolishes the effect (Boulant et al., 1994). Impairment of LES sphincter function can lead to gastroesophageal reflux disease (Zerbib et al., 1998). Thus, through a NO-mediated pathway, CCK plays a role in preventing acid reflux to the esophagus in the intestinal phase of the digestive process (Figure 5). Duodenal nutrient infusion initiates relaxation of the proximal stomach. Therefore, it has been hypothesized that a postprandial duodenogastric feedback, including release of CCK, is involved in the control of gastric accommodation. Avoiding the entrance of nutrients in the small bowel eliminates nutritional feedback and hampers gastric accommodation in a nutrient tolerance test (Demarchi et al., 2004; Carbone et al., 2019). Administration of orlistat, a selective lipase inhibitor, decreases plasma CCK levels but does not affect gastric accommodation during a satiety drinking test. Whether CCK actually induces the drop in fundic tone in a NO-dependent fashion has yet to be elucidated (Kindt and Tack, 2006). In dogs, gastric contractility and emptying are suppressed after treatment with a competitive NOS inhibitor (Tanaka et al., 2005). CCK induces contractions of the smooth muscle of the colon by interacting with the CCK1R in humans (Morton et al., 2002). By contrast, CCK2Rs are found to mediate the inhibitory actions of CCK on motor activity in human distal colon muscles strips and evidence exists that this action is NO-dependent, which is also found in rat *in vivo* experiments and *in vitro* studies in canine colon cells (Publicover et al., 1993; Takahashi et al., 2005; Fornai et al., 2007).

The CCK-NO system may respond to increased blood flow in the gastrointestinal tract with digestion of nutrients. In isolated bovine cerebral and mesenteric arteries, CCK induces neurogenic vasodilatation by acting on the CCK2R, and this is nNOS dependent (Sánchez-Fernández et al., 2003; Sanchez-Fernandez et al., 2004). This suggests that CCK stimulates nNOS activity and thus NO release (Ruiz-Gayo et al., 2006). CCK mediated splanchnic vasodilatation is also prevented by NOS inhibition (Lovick, 2009). Intravenous administration of CCK8 results in a decrease in the mesenteric vascular resistance and leads to vasodilatation of the pancreatic artery in dogs (Thulin and Olsson, 1973; Chou et al., 1977; Premen et al., 1985; Doi et al., 1990). Moreover, it is likely that the increased blood flow by NO also results in augmented CCK-induced pancreatic secretion of fluid, bicarbonate, and protein in both *in vivo* and -*vitro* dog and rat models (Konturek et al., 1993; Patel et al., 1995; Jyotheeswaran et al., 2000). Isolated canine pancreatic arteries, which lack a full innervation, are less sensitive to CCK, which suggests the involvement of neurons (Ruiz-Gayo et al., 2006). The CCK induced vasodilation in the pancreas mainly affects the exocrine part and is less prominent for the endocrine part. It is suggested that the postprandial CCK response improves pancreatic enzyme secretion, while there is a possibility that insulin release toward its targets is enhanced in anesthetized rats (Iwase et al., 2003). Exogenous and endogenous CCK induces gastric mucosal hyperemia in response to sensory nerve stimulation by luminal noxious stimuli such as ethanol or bile (Evangelista et al., 1987; Evangelista and Maggi, 1991; Stroff et al., 1994; Konturek et al., 1995a; Sánchez-Fernández et al., 2003; West et al., 2003). Inhibition of NOS reduces the ability of CCK to augment gastric blood flow *in vivo* in rats (Stroff et al., 1994; Konturek et al., 1995c; Heinemann et al., 1996). In one *in vivo* study, CCK increases Ca^2+^-dependent NOS activity, primarily via eNOS and not via iNOS in rats (West et al., 2003). Controversy exists which CCK receptor is involved in the process of gastric hyperemia and blood flow, as both the CCK1R (Brzozowski et al., 1999; West et al., 2003) and CCK2R (Heinemann et al., 1995) have been suggested in anesthetized rats. Of notice, both receptor subtypes are able to activate the NO/cGMP pathway (Aras and Ekström, 2006; Dufresne et al., 2006).

NO is involved in the peptide regulation of feeding in the central nervous system, including CCK’s. The intake-reducing actions of endogenous CCK depend upon the vagal nerve and the dorsal vagal complex in a rat *in vivo* model (Sullivan et al., 2007). In mice, administration of CCK inhibits food intake, but fails to do so in nNOS-knockout mice (Morley et al., 2011). CCK is found to co-exist with NOS in rat hypothalamus suggesting that NO works in conjunction with CCK to regulate feeding behavior at a central level (Yamada et al., 1996; Morley et al., 2011). Besides the hypothalamus, CCK and NO are functionally involved in regulatory neurotransmission in other parts of the rat brain including the cortico-striato-pallidal circuitry (Ferraro et al., 2003).

Little is known about the role of CO or H_2_S in the CCK up- or downstream pathways. In rat pancreatic acinar cells, both H_2_S and CO govern Ca^2+^ homeostasis and CO was shown to intervene with the CCK8-induced enzymatic secretions. They stimulate NOS expression and NO production in these cells, although blocking the NO-pathway has no effect on the increased Ca^2+^ levels for CO, while for H_2_S the effect is largely abolished (Moustafa and Habara, 2014a; Moustafa and Habara, 2014b).

CCK also has anti-inflammatory actions, possibly by reducing vascular and macrophage iNOS-derived NO production in rat *in vivo* models (Saia et al., 2013; Zuelli et al., 2014). In lipopolysaccharide (LPS)-stimulated peritoneal macrophages, the inhibition of iNOS mRNA expression by CCK is accompanied by inhibition of the NF-*κ*B signaling pathway and an increase in intracellular cAMP content, activation of the protein kinase A (PKA) pathway and up-regulation of CCK1R in anesthetized rats (Saia et al., 2014). The anti-inflammatory effect of CCK may regulate the excessive macrophage activation effect of LPS contained in food. CO has also been suggested as a potential treatment for acute pancreatitis. Serum CCK8 levels are remarkably increased after an LPS-induced inflammation at the site of the lungs. The protective effect of CCK8 is abolished after the treatment with a HO-1 inhibitor, suggesting the *in vivo* involvement of CO in rats (Huang et al., 2004). Similar results are found for H_2_S. The lungs are sensitive organs for an endotoxic shock and are therefore more studied. Blocking the CSE activity worsens the acute lung injury (Moustafa and Habara, 2014a). CCK8 induces CSE activity and attenuates the injury in an anesthetized rat model (Tian et al., 2017). More research is needed for other organs such as the pancreas. Under healthy circumstances, HO-1 and HO-2 are not expressed in the exocrine part, while HO-1 expression is induced during a period of inflammation (Moustafa and Habara, 2014a). CCK8 can have an impact on multiple organs by reducing the proinflammatory cytokines in the circulation in an *in vivo* rat model (Ling et al., 2001). However, there are various issues to consider; e.g. whether CCK also has an organ-protecting effect in humans, whether CCK exhibits an organ-protecting effect at physiological concentrations, and why CCK, which is secreted after eating, has an organ-protecting effect.

HO-2 is expressed in myenteric neurons, in neuronal cell bodies and in nerve fibers in the gallbladder. Together with NO and VIP, H_2_S functions as a neurotransmitter to induce muscle relaxation. Exogenous CO induces a relaxation in dog gallbladder strips, and a specific HO-inhibitor attenuates the neurally mediated relaxation of an electrical field stimulation, which implies a role for endogenous CO. Whether it is involved in the actions of CCK, has yet to be elucidated (Alcon et al., 2001).

## Secretin

Secretin is produced in S-cells of the duodenum and jejunum. It is involved in the small bowel pH regulation through inducing bicarbonate secretion and suppressing gastric acid secretion. The latter is mediated by SST and prostaglandin. In addition, secretin induces gastric relaxation, delays gastric emptying and induces secretion of pancreatic enzymes (i.e., amylase and lipase). The pH neutralization is important for the activity of these enzymes (Hacki, 1980; Chey and Chang, 2003). The main trigger for secretin release is acid delivered into the duodenal lumen. Digested products of fat and proteins, bile acid and herbal extracts also contribute to its release (Chey and Chang, 2001). Like CCK, the release of secretin along with pancreatic exocrine secretion is controlled through a feedback regulatory mechanism mediated by pancreatic proteases (Green and Lyman, 1972). The expression of the secretin receptor has been demonstrated in a number of organs including the brain (cerebellum, hippocampus and central amygdala) of humans and rodents, pancreas, stomach, kidney as well the biliary epithelium in the liver (Afroze et al., 2013). Besides the important roles of secretin in increasing hepatic and pancreatic secretion of sodium bicarbonate and inhibition of gastrin release, secretin has additional functions. In stomach, secretin stimulates gastric pepsin secretion and suppresses gastric emptying, which facilitates digestion of proteins in duodenum (Vagne and Andre, 1971; Sanders et al., 1983). In the kidney, secretin plays a role in increasing urinary volume and bicarbonate excretion in normal human subjects, which helps to maintain body water homeostasis (Baron et al., 1958; Viteri et al., 1975). In the rodent brain, secretin can act as a central nervous system peptide neurotransmitter (Yang et al., 2004; Nishijima et al., 2006; Yamagata et al., 2008). For this reason, secretin has gained substantial attention as a putative treatment approach to autism and affective disorders, but this remains an area of ongoing controversy (McQueen and Heck, 2002; Boismenu et al., 2004). Secretin also affects the cardiovascular system (Chou et al., 1977). In human, infusion of secretin in both controls and patients with decreased left ventricular function, increased cardiac output, stroke volume and decreased systemic vascular resistance (Gunnes et al., 1983; Gunnes and Rasmussen, 1986).

Although NOS is expressed in 40% of the myenteric plexus, VIP and not NO, is involved in the gastric relaxation induced by secretin *in vivo* in rats (Lu and Owyang, 2009). Administration of NO-generating compounds does not affect the release of SST, which lays downstream of secretin’s suppression of gastric acid release in isolated rat synaptosomes (Kurjak et al., 1996).

**FIGURE 6:**
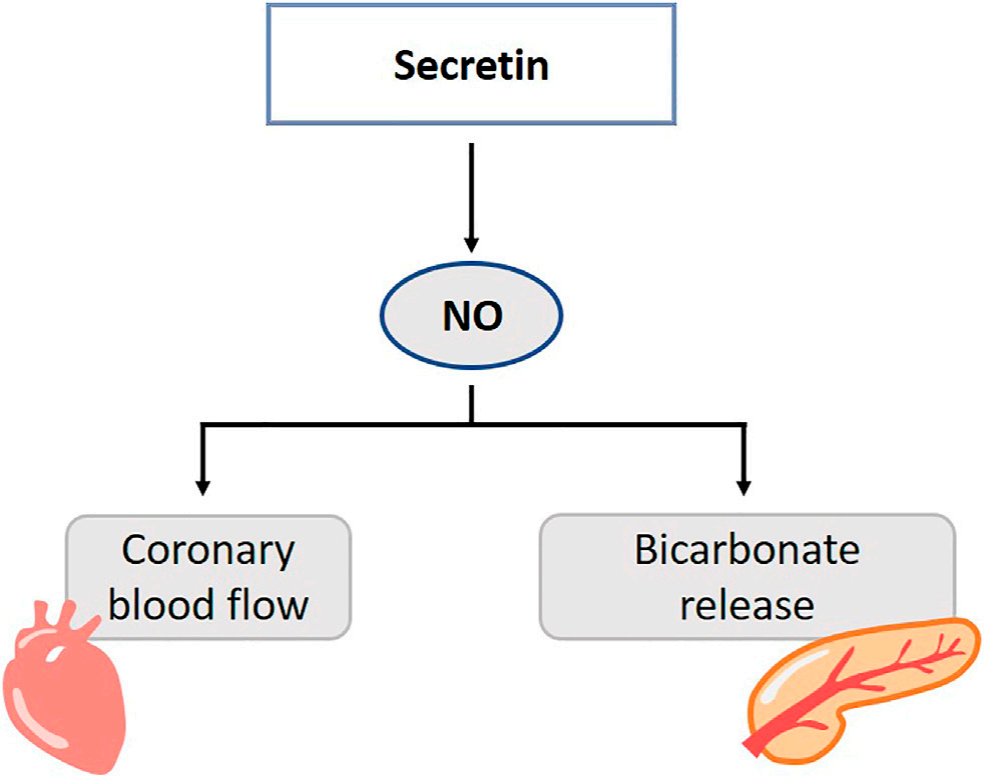
Role of gasotransmitters on secretin’s physiological functions. Solid lines are clear evidence–based on blockers and substrate administration. NO mediates the action of secretin on the exocrine pancreas. Release of NO is involved in the secretin-induced increase in coronary blood flow. NO, Nitric oxide.

In rats and cats, inhibition of NO synthesis in the pancreas results in a dose-dependent decrease of the secretin-stimulated, bicarbonate secretion (Patel et al., 1995; Jyotheeswaran et al., 2000; Konturek et al., 1997). This was restored by l-arginine and NO-inhibition did not affect endogenous secretin plasma levels (Jyotheeswaran et al., 2000). It was suggested that NO mediates the action of secretin on the exocrine pancreas, probably through neuronal pathways, and not the release of secretin (Kirchgessner et al., 1994; Chey and Chang, 2014) (Figure 6). Little is known about the role of CO and H_2_S in the secretin pathways. Duodenal acidification is sensed by capsaicin-sensitive sensory neurons, which in their turn induce NO. NO stimulates bicarbonate release via cGMP-dependent kinase I, which is suggested to be responsible for 70% of the acid-induced bicarbonate release. Endo- or exogenously generated H_2_S and CO stimulate bicarbonate secretion in the duodenum of rodents. The stimulatory action of NO and CO is mediated by endogenous prostaglandins, while that of H_2_S is mediated by prostaglandins and NO and also involves sensory neurons (Seidler, 2013; Mard et al., 2016a). Direct evidence of the involvement of secretin in this pathway is lacking. These results rather raise the impression that secretin function and gasotransmitters are not entangled.

In the healthy and diabetic rat excess or lack of NO changes the blood pressure, cardiac and coronary effects of secretin (Sitniewska et al., 2000a; Sitniewska et al., 2000b; Sitniewska et al., 2002). In pigs, release of NO is involved in the secretin-induced increase in coronary blood flow (Grossini et al., 2011) (Figure 6). l-NAME decreases vascular permeability of the left ventricle and atria in intact and diabetic rats. Secretin elevates systolic and diastolic blood pressure in *in vivo* rats, but lack an effect on vascular permeability. Secretin abolishes the NOS-inhibition by l-NAME, and restores microcirculation function (Sitniewska and Wiśniewska, 2001).

## Motilin

Since recent studies showed that motilin plays a role in the control of hunger and regulation of food intake in humans in both health and disease, the peptide has attracted more attention (Deloose et al., 2019). It stimulates gastrointestinal motility in several mammals, but not in rodents (He et al., 2010), through direct action on the smooth muscle cells and/or activation of the cholinergic or noncholinergic neural pathways in the isolated chicken gut (Kitazawa et al., 2002). Motilin is released by enteroendocrine M-cells in the proximal duodenal region (Wierup et al., 2007). In the fasting state, plasma levels of motilin fluctuate in parallel with phase III contractions of the migrating motor complex (MMC), waves of electrical activity that sweep through the intestines in a regular cycle during fasting (Deloose et al., 2012). Changes in the luminal environment of the duodenum, dietary components, and hormones on motilin release have been reported to affect motilin release (Deloose et al., 2019). Acidification of the duodenum and bile acids stimulate the release of motilin when foods move into the duodenum, and alkalization of the duodenum suppresses it after pancreatic juice secrets into the duodenum in humans (Collins et al., 1981; Svenberg et al., 1984; Qvist et al., 1995). The release of motilin after a meal depends on the nutrient composition of the meal. A small reduction occurs after the administration of glucose, but lipids markedly increase the release of motilin (Mitznegg et al., 1976). Other hormones such as insulin, SST, and secretin reduce motilin levels (Mitznegg et al., 1977; Jenssen et al., 1984; Jenssen et al., 1986). In the gastrointestinal tract, motilin increases the pressure of LES, initiates gastric phase III contractions of the MMC, stimulates gastric emptying, inhibits gastric accommodation and increases rectal compliance in humans (Domschke et al., 1976; Bormans et al., 1987; Peeters et al., 1992; Kamerling et al., 2003; Cuomo et al., 2006). Insulin secretion and gallbladder emptying are both increased by motilin in humans and dogs (Luiking et al., 1998; Suzuki et al., 1998; Suzuki et al., 2003). Finally, motilin also activates regions in the brain involved in the homeostatic and hedonic regulation of food intake in humans (Zhao et al., 2018). These physiological effects of motilin have not been completely elucidated, however it is hypothesized that motilin helps to clean the stomach to receive the next food in the fasted state (Itoh, 1997). This may also explain why motilin causes hunger stimulation (Tack et al., 2016).

**FIGURE 7:**
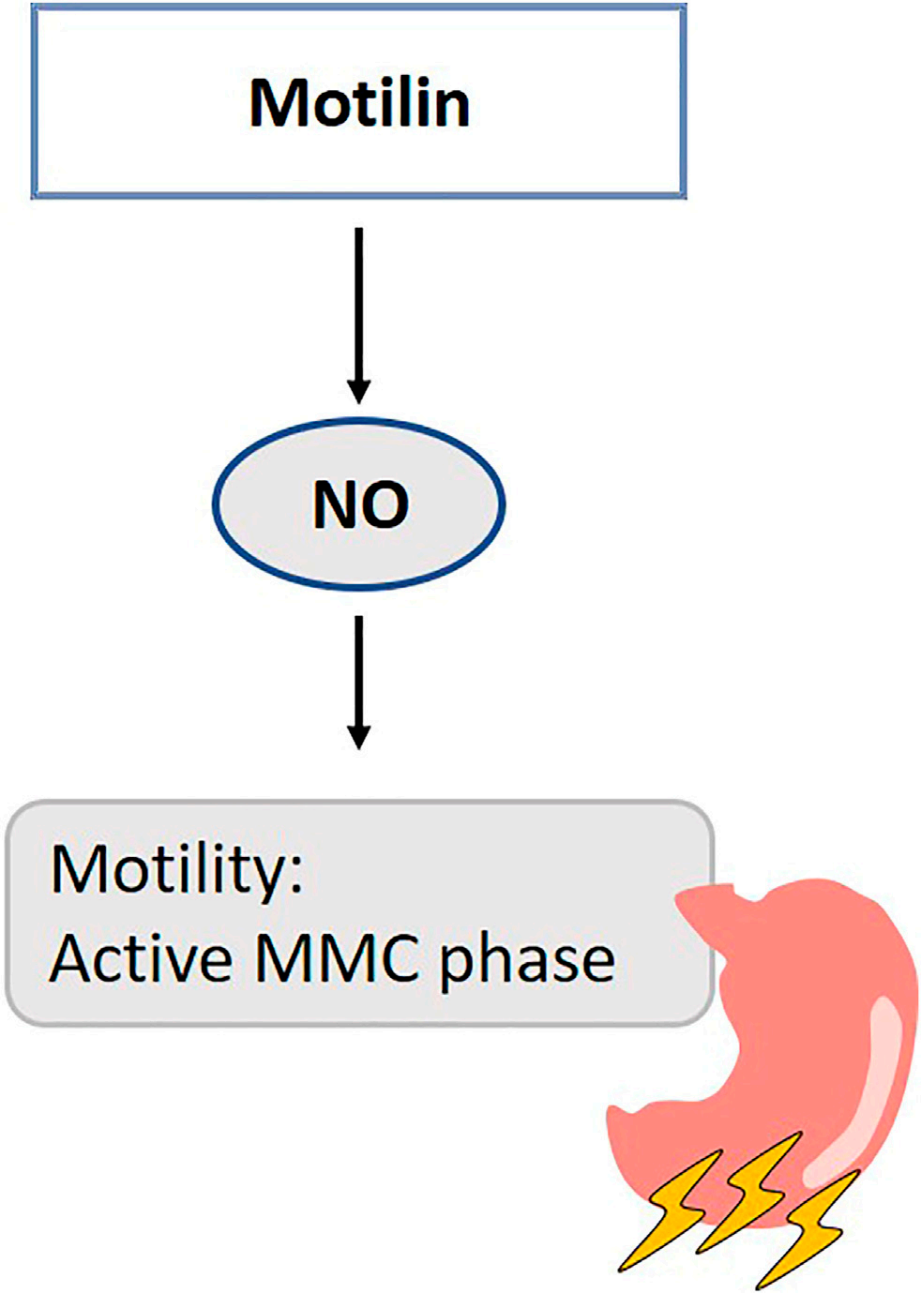
Role of gasotransmitters on motilin’s physiological functions. Solid lines are clear evidence–based on blockers and substrate administration. NO modifies the contractile response of motilin in the antral part of the stomach during the interdigestive state. NO, Nitric oxide; MMC, Migrating motor complex.

Motilin mediates the contractility of smooth muscles in the stomach through the nervus vagus and/or directly through its receptors at this site in human (Itoh, 1997). NO functions as a neurotransmitter for non-adrenergic, non-cholinergic inhibitory neurons and has been suggested to modify the contractile response of motilin in the stomach. NO synthase inhibition potentiates the contractile response of motilin in the chicken proventriculus through reduction of endogenous NO-mediated presynaptic inhibition on neural acetylcholine release (Kitazawa et al., 2002) (Figure 7). Thirty minutes after NOS-inhibition, phase 3-like contractions are initiated in the stomach and duodenum, simultaneous with motilin release in fasted dogs (Sarna et al., 1993; Mizumoto et al., 1997). After this period of activity, the MMC cycle is disrupted, which is suggested to be the consequence of blocked NANC nerves (Sarna et al., 1993). The endogenous motilin release is controlled via a cholinergic pathways independent of the vagus nerve (Mizumoto et al., 1997). In human, L-NMMA induces a premature duodenal phase III within several minutes after administration. Motilin plasma levels were not affected (Halim et al., 2015). These observations led the authors to suggest that L-NMMA provides a direct inhibition of nNOS in the myenteric plexus, independent of endocrine regulators (Halim et al., 2015). The mechanism of action of motilin in dogs is thought to be different from that in humans, so these results need to be interpreted carefully (Peeters et al., 1988). Besides their expression in the stomach and duodenum, motilin receptors are also abundantly present in the central nervous system, in particular the hippocampus in rats (Lange et al., 1986; Guan et al., 2003a; Liu et al., 2005) where there is indirect evidence for interaction between NO and motilin (Lu et al., 2015). Nevertheless, the consequences of this finding and the exact mechanisms underlying interaction between motilin and NO remain to be elucidated.

Both motilin (or the motilin receptor agonist erythromycin) and H_2_S donors are considered prokinetic drugs for gastric emptying (Peeters et al., 1992; Mard et al., 2016b). The H_2_S studies were performed in rodents, which do not have a functional motilin or motilin receptor gene. This hypothesis should be worked out in human *ex vivo* samples from biopsies.

## Ghrelin

Ghrelin is released by P/D1-cells in humans or X/A-like-cells in rats, which are mainly situated in the stomach, and more restricted in the duodenum and pancreas (Kojima et al., 1999; Date et al., 2000). Ghrelin-*o*-acyltransferase enzyme activates about 7% of the total des-acyl ghrelin production in humans (Marzullo et al., 2004), and stores both forms until secretion is triggered. Ghrelin peaks in the plasma right before a spontaneous meal initiation, and decreases drastically after (Cummings et al., 2001). While lipid loads have no prominent effect on ghrelin, glucose and ingested proteins cause a steep decrease (Foster-Schubert et al., 2008). Different factors influence ghrelin baseline values in the plasma. In the fasted state, it is negatively correlated with BMI as a result of the positive energy balance (Shiiya et al., 2002). In addition, the more *H. pylori* thrives and gastric tissue degrades, the lower the ghrelin plasma levels (Tatsuguchi et al., 2004). Lastly, hormones such as testosterone and growth hormone (GH) releasing hormone stimulate ghrelin levels, while insulin, GH, interleukin-1, leptin, SST, urocortin-1, thyroid hormone, CCK, peptide YY (PYY) and melatonin decrease these (Nonogaki, 2008). Ghrelin was first discovered as the growth hormone-releasing peptide and binds the growth hormone secretagogue-receptor 1a (GHSR1a) (Kojima et al., 1999), which is expressed in various regions of the brain (in the pituitary gland for growth hormone secretion, but also in the feeding control regions of the hypothalamus), endothelial, myocardial, pancreatic, renal, adrenal tissues and immune cells (Gnanapavan et al., 2002; Volante et al., 2002; Dass et al., 2003; Tortorella et al., 2003). Besides stimulating GH release and food intake, ghrelin stimulates gastric acid secretion, gastrointestinal motility, modulates energy balance, taste sensation, stress and anxiety, glucose metabolism and has cardiovascular effects (Nakazato et al., 2001). Due to the multiple effects of ghrelin, it is considered a promising therapeutic target for various diseases, including cancer-related cachexia (Garcia et al., 2013). For a comprehensive overview of the effects of ghrelin and the consequences of administration to human, we refer to the literature (Garin et al., 2013; Collden et al., 2017).

Ghrelin acts on the pituitary GHSR to promote the release of GH. In the porcine anterior pituitary, ghrelin activates the NOS/NO/GC/cGMP signaling pathway, essential for the stimulation of somatotropes (Rodríguez-pacheco et al., 2005). On the other hand, the activation of GHSR1a induces GH release through enhanced phospholipase C activity, protein kinase C and intracellular calcium mobilization in pigs (Chen et al., 1996).

Administration of ghrelin into the arcuate and paraventricular nuclei of the hypothalamus has a potent orexigenic effect and increases body weight by suppressing energy expenditure in rats (Currie et al., 2010). Ghrelin secreted from the stomach is thought to transmit hunger signals via the afferent vagal nerve to the center, and promotes feeding behavior by dual control of activation of anorexic nerves - neuropeptide Y (NPY) and agouti-related protein (AgRP) - and inhibition of feeding behavior suppressive pro-opiomelanocortin (POMC) nerves in rats (Shintani et al., 2001; Kojima and Kangawa, 2005). In the rodent hypothalamus, both NOS and GHSR1a mRNA are expressed (Ng et al., 1999; Zigman et al., 2006). In mice, the intracerebroventricular administration of l-NAME results in an attenuated ghrelin stimulants on food intake (Gaskin et al., 2003). Moreover, in NOS knockout mice the administration of ghrelin does not result in increased food intake (Morley et al., 2011). NO is reported as a signaling molecule in neurons, neuropeptide regulation and stimulates feeding in many species (Morley et al., 2011; Han et al., 2013). Although a direct NO-mediated pathway by ghrelin has not been proven yet, these data suggest that reactive oxygen species may regulate food intake through modulation of the NO-bioavailability.

**FIGURE 8:**
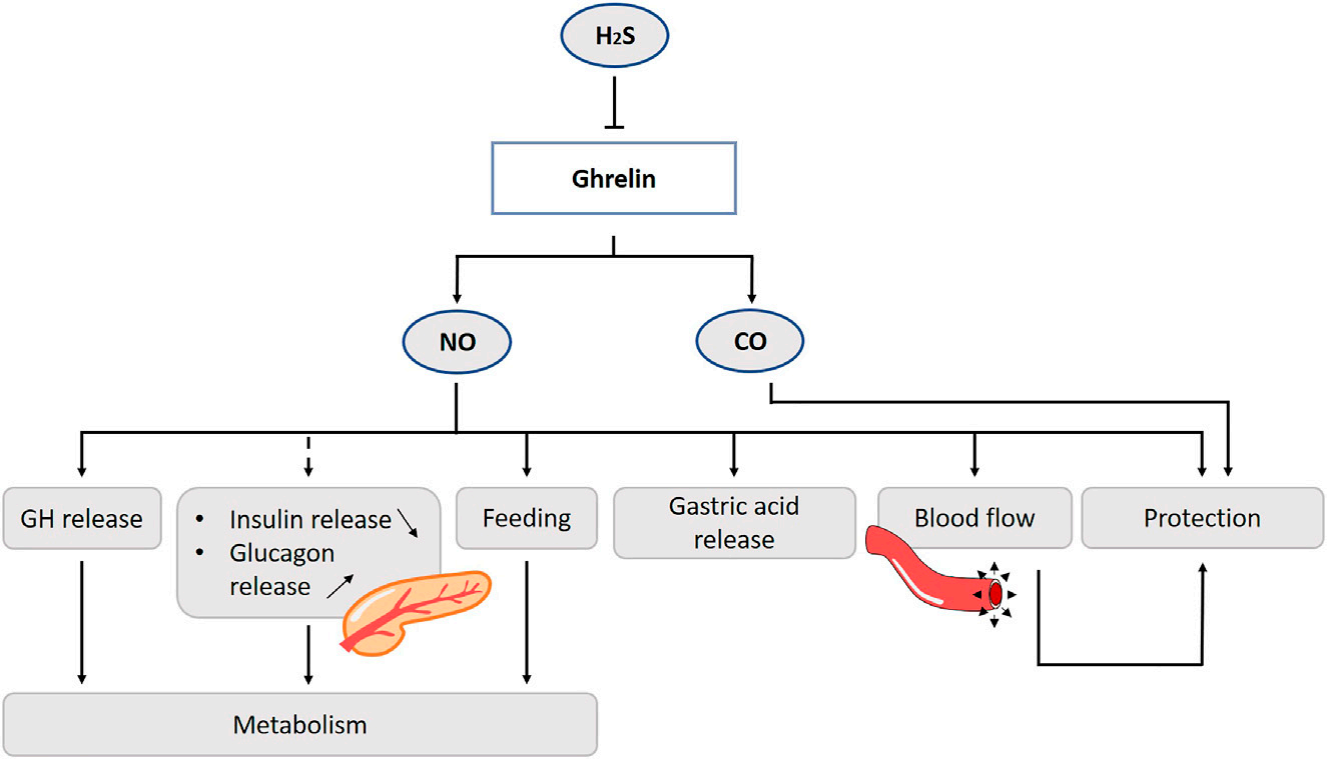
Role of gasotransmitters on ghrelin release and its physiological functions. Solid lines are clear evidence–based on blockers and substrate administration. Dotted lines represent first evidence for the presence of the pathway. The amino acid l-cysteine, which is a H_2_S donor, suppresses ghrelin release from the stomach. Ghrelin promotes the release of GH via activation of the NOS/NO/GC/cGMP-signaling pathway. Ghrelin also promotes feeding behavior and stimulates gastric motility and acid secretion via the NO-pathway. No direct evidence is found to date for the involvement of NO in the ghrelin-glycemic effect. Ghrelin protects the gastric mucosa, by stimulation of the blood flow and hyperemia mediated by NO. CO is also involved in the ghrelin-mediated gastroprotection. NO, Nitric oxide; CO, Carbon monoxide; H_2_S, Hydrogen sulfide; GH, Growth hormone; NOS, Nitric oxide synthase; GC, Guanylyl cyclase; cGMP, Cyclic guanosine monophosphate.

Ghrelin release is suppressed for a long time after a high protein meal (Foster-Schubert et al., 2008). The amino acid l-cysteine, which is a H_2_S donor, suppresses ghrelin release from the rat stomach, and reduces appetite. On the other hand, direct inhibition of H_2_S synthesis stimulates ghrelin secretion (Figure 8). Ghrelin cells co-localize with H_2_S producing enzyme CSE. There seems to be an interplay between these two factors, which provides us of new ideas for treatments e.g., a diet enriched of H_2_S precursors or sulfur-rich prebiotics to affect ghrelin levels and appetite in mice (Slade et al., 2018).

One of the first established functions of ghrelin were the stimulation of gastric motility and acid secretion (Masuda et al., 2000). Ghrelin is thought to exert these effects primarily via pathways mediated by the vagus nerve (Date et al., 2002). This involves activation of GSHR1a on vagal afferent neurons. In rats, exogenous ghrelin administration increases gastric acid output, mucus content and total plasma nitrite levels, while these effects are inhibited by l-NAME (Bilgin et al., 2008).

In animal studies, ghrelin has a protective effect of the gastric mucosa. In an ischemia/reperfusion experiment using rats, the addition of ghrelin accelerates healing in part by stimulating blood flow and hyperemia mediated by NO (Konturek et al., 2006). Of interest, inhibition of NOS by l-NAME and sensory denervation by capsaicin, but not vagotomy, prevents ghrelin’s gastroprotective effects of ethanol-induced ulcers in rodents (Sibilia et al., 2003). HO-1, and thus presumably CO, is also involved in the ghrelin-mediated gastroprotection (Allam and El-Gohary, 2017) (Figure 8).

Ghrelin has well-documented cardiac beneficial effects, including protection from ischemia/reperfusion injury, attenuation of left ventricular remodeling following myocardial infarction, and improvement of left ventricular function (Virdis et al., 2016). At the level of blood vessels, ghrelin has a significant impact on vascular function. In endothelial cells and in intact vessels, ghrelin stimulates the phosphorylation of eNOS at Ser-1177, and not Thr-495 which would inactivate the activity *in vitro* (Iantorno et al., 2007; Xu et al., 2008; Dudzinski et al., 2006). This action of ghrelin involves signaling through GHSR1a, PI 3-kinase, Akt, and eNOS (Iantorno et al., 2007). In addition, there is evidence that the AMP-activated protein kinase (AMPK) is also a mediator for ghrelin activation of eNOS (Xu et al., 2008). Using a GHSR1a knockdown model abolishes the ghrelin influence on endothelial cells (Chen et al., 2013). The activation of eNOS by ghrelin potentiates the NO-mediated relaxation of vascular smooth muscle and diminishes the production of reactive oxygen species in reaction to endothelial injury (Iantorno et al., 2007; Xu et al., 2008; Hedayati et al., 2009) (Figure 8). In patients with metabolic syndrome, administration of ghrelin leads to an increase in NO bioactivity and improved endothelial function (Tesauro et al., 2005; Tesauro et al., 2009). It should be noted that some studies suggest that the vasomotor actions of ghrelin are NO-independent in humans and rats (Okumura et al., 2002; Wiley and Davenport, 2002; Shinde et al., 2005). In healthy men, an intra-arterial bolus of ghrelin increases forearm vasodilatation in a dose-dependent manner but this effect is NO-independent (Okumura et al., 2002). Here, the administration of ghrelin does not alter either plasma insulin-like growth factor-1 (IGF-1) or the NO second messenger, cGMP, but decreases plasma norepinephrine. These observations might implicate the involvement of other mechanisms, i.e., the autonomic nervous system. On the other hand, in aforementioned experiment the L-NMMA was only infused for 5 min leaving the possibility that eNOS activity is incompletely inhibited.

It is conceivable that the inhibitory effect of ghrelin on insulin and stimulatory on glucagon release are mediated by nNOS in pancreatic rat cells (Qader et al., 2005). In the vascular endothelium, ghrelin and insulin share the PI 3-kinase/phosphoinositide-dependent kinase-1/Akt/eNOS signaling pathway in human (Montagnani et al., 2002; Iantorno et al., 2007). This might account for some of the beneficial effects of ghrelin on metabolic and cardiovascular disease. However, there is controversy about whether ghrelin stimulates or suppresses insulin secretion. This may be due to differences in reactivity depending on animal species or experimental design, so caution should be taken in the interpretation (Dezaki et al., 2004).

Ghrelin exhibits antioxidant effects in many organs, such as heart, pancreas and lung (Suzuki et al., 2011). In rats, injection of ghrelin results in enhanced hepatic expression of antioxidant enzymes (Dobutovic et al., 2014). Together with observations of increased plasma ghrelin concentrations during systemic oxidative stress (Okumura et al., 2002; Bouros et al., 2006; Guzik and Harrison, 2006; Liu et al., 2006) this lead to the suggestion ghrelin could have positive effects on oxidative injury (Suzuki et al., 2011). In rats, treatment with ghrelin after occlusion of the middle cerebral artery results in a decrease in cerebral TNF-α, IL-6, neutrophil trafficking, matrix metalloproteinase 9, nitrotyrosine, and nNOS gene expression. Ghrelin’s protective effect is abolished in vagatomized rats, suggesting the involvement of the vagus nerve (Cheyuo et al., 2011).

## Glucagon-Like Peptide 1

Glucagon-like peptide 1 (GLP-1) is released from L-cells, situated in the ileum and colon (Eissele et al., 1992). In response to the ingestion of dietary carbohydrates or fat, GLP-1 is released and functions as incretin and anorexigenic hormone (Layer et al., 1995). Non-nutrient stimulators of GLP-1 release are the neuromodulators acetylcholine and gastrin-releasing peptide. Peripheral hormones that participate in energy homeostasis, such as leptin, are implicated in the regulation of GLP-1 release (Lim and Brubaker, 2006). Leptin stimulates GLP-1 release from the human and rodent L-cell, and this effect is abolished in leptin-resistant obese mice (Anini and Brubaker, 2003). GIP regulates proglucagon-derived peptide secretion in both *in vivo* and *in vitro* rat models (Lim et al., 2009). In the pancreas, GLP-1 enhances insulin release in the presence of high glucose levels in the blood and inhibits glucagon release (Holst, 2007). It suppresses gut motility, resulting in slower entry of the carbohydrates into the small bowel. GLP-1 increases satiation and therefore governs meal size. Numerous studies have shown that GLP-1 levels after a meal are reduced in subjects with type 2 diabetes and obesity (Toft-Nielsen et al., 2001; Anini and Brubaker, 2003). GLP-1 agonists, such as liraglutide, are used as a treatment for these pathologies (Meece, 2017). GLP-1 was discovered in 1979 (Lund, 2005), and after 30 years of research, it has proven to have a broad range of functions, being an incretin, an anorexigenic peptide, decreasing gut motility, but also received more attention recently for its cardiac functions (Lim and Brubaker, 2006; Sun et al., 2015). Many of these functions are in overlap with those of NO, H_2_S and CO.

**FIGURE 9:**
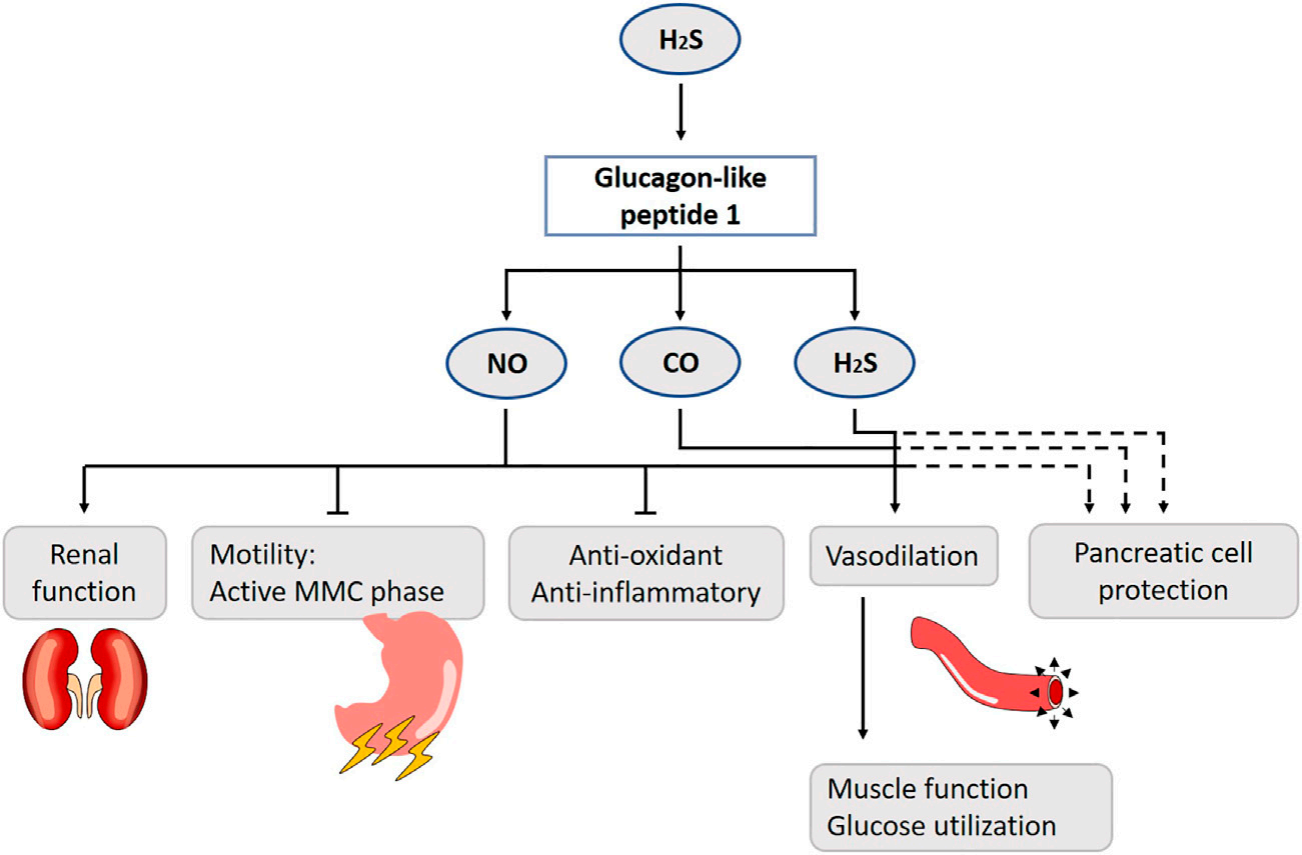
Role of gasotransmitters on glucagon-like peptide 1 (GLP-1) release and its physiological functions. Solid lines are clear evidence–based on blockers and substrate administration. Dotted lines represent first evidence for the presence of the pathway. H_2_S stimulates GLP-1 release. The suppression of gut motility, reduction of nephropathy risk factors, increase of muscle microvasculature, reduction of the number of reactive oxygen species and production of vasoconstrictive mediators are GLP-1 effects mediated by NO. H_2_S and CO also contribute to the vasodilation. It has been suggested that the gasotransmitters play a protective role to govern pancreatic function in case of glucotoxicity. NO, Nitric oxide; CO, Carbon monoxide; H_2_S, Hydrogen sulfide; MMC, Migrating motor complex.

H_2_S is produced in the colon by sulphate-reducing bacteria, and stimulates GLP-1 release. Considering its short half-life time, it will stimulate nearby L-cells in the colon rather than ileal L-cells after transport in the plasma (Pichette et al., 2017). Four weeks treatment with chondroitin sulfate increases H_2_S production, enhances GLP-1 and insulin secretion, improves oral glucose tolerance and reduces food consumption (Pichette et al., 2017) (Figure 9). Although NOS is expressed in brush cells - luminal sensors adjacent to enteroendocrine cells -, no direct evidence for NO or CO’s involvement has been found (Sternini et al., 2008).

GLP-1 slows gastric emptying, inhibits the active antral MMC phases and prolongs small bowel transit time. It is able to cross the blood-brain barrier (Kastin et al., 2002) and a high density of GLP-1 receptors is present in the vagal circuits in the brainstem (Shimizu et al., 1987; Göke et al., 1995). As such, one could hypothesize that peripherally released GLP-1 functions as a classic hormone by migrating to the central nervous system where it acts on the brainstem. It seems more likely that the brainstem vagal circuits are affected by GLP-1, after activation of gastro-intestinal stimuli (Shimizu et al., 1987). A 1–2 pmol/kg^−1^ GLP-1 infusion prolongs the MMC cycle length and abolishes its active antral phase by NO. With a dose of 10 pmol/kg^−1^, the effect becomes NO-independent in conscious rats (Tolessa et al., 1998; Tolessa et al., 2001). A GLP-1 analogue, LY315902, also decreases gut motility via the NO-pathway in mice (Rotondo et al., 2011).

GLP-1 is mainly known as an incretin. Literature about the role of gasotransmitters in the GLP-1-insulin release is contradictory. At first sight, it seems like the gasotransmitters have a protective role to govern pancreatic function in case of glucotoxicity. Under normal circumstances, the inducible HO-variant is not present in rat beta cells, although in obese rats HO-1 seems to be upregulated (Lundquist et al., 2003). Similar results are shown for iNOS, which is normally only expressed as response to an inflammatory agent, and clearly, an episode of hyperglycemia (20 mmol/L) is considered stressful for the pancreatic beta cell. GLP-1 induces a cAMP/PKA-pathway inhibiting the expression of iNOS, and prevents beta cell apoptosis (Jimenez-Feltstrom et al., 2005). For H_2_S, only CBS is expressed under normal glucose levels in the mouse beta cells, while CSE is induced by hyperglycemia (Beltowski et al., 2018). H_2_S inhibits insulin secretion by the activation of ATP-sensitive potassium channels, but only in the presence of glucose. Furthermore, it affects beta cell survivability, although the literature is inconsistent on whether it induces apoptosis or in contrast protects these cells (Pichette and Gagnon, 2016; Beltowski et al., 2018). Diabetic animal models or patients have lower H_2_S blood concentrations (Suzuki et al., 2017). More research should be performed using H_2_S-donors as treatment. H_2_S has roles in glycemia control beyond insulin, as it plays a role in the liver, muscles and even fat tissue, but this is discussed in more detail in the literature (Pichette and Gagnon, 2016; Beltowski et al., 2018).

GLP-1 receptor agonists have hypotensive actions (Sun et al., 2015). The potential mechanism may be directly mediated via GLP-1 receptor activation on blood vessels and kidney, including improvement of endothelial function, vasodilatation, and natriuresis (Gutzwiller et al., 2004; Nyström et al., 2004; Lorber, 2013; Sun et al., 2015). GLP-1 receptors are expressed in the arteries, glomeruli, and proximal tubules of the kidney (Schlatter et al., 2007; Crajoinas et al., 2011). In human and rodent studies, activation of these receptors results in diuresis, natriuresis and renal vasodilatation (Moreno et al., 2002; Thomson et al., 2013). Several studies established that the effects of GLP-1 reduce risk factors of nephropathy, including hypertension and albuminuria, and lead to renoprotection. As an underlying mechanism, stimulation of the GLP-1 receptor by the GLP-1 agonist exenatide results in an increased glomerular filtration rate, urinary flow rate and urinary prostaglandin E2 and NO degradation products. Pre-treatment with L-NMMA reduces urinary NO degradation products and the impact of exenatide on glomerular filtration rate and urinary flow rate by 50%, implying that NO mediates a part of the renal hemodynamic actions of exenatide in anesthetized rats (Thomson et al., 2017).

Several studies demonstrated that administration of GLP-1 analogues increases NO production in endothelial cells and improves endothelium-dependent vasodilatation in rats (Han et al., 2012; Sélley et al., 2014; Green et al., 2008) (Figure 9). In overnight-fasted male rats, continuous infusion of GLP-1 acutely increases muscle microvasculature, which is associated with increased muscle glucose utilization, plasma concentrations of NO, muscle interstitial oxygenation and muscle insulin clearance/uptake (Chai et al., 2012). Co-infusion of l-NAME abolishes this effect, suggesting that GLP-1 acts via a NO-dependent mechanism. From a mechanistic point of view, GLP-1 increases PKA activity and stimulates eNOS’s phosphorylation at Ser(1,177) cultured endothelial cells (Dong et al., 2013). The incubation of human umbilical veins with liraglutide promotes eNOS expression (Dai et al., 2013). Furthermore, the dipeptidyl peptidase-4 (DPP-4) inhibitor sitaglipitin is shown to enhance eNOS phosphorylation through the cAMP/PKA pathway by augmenting GLP-1 activity in human coronary artery endothelial cells (Matsubara et al., 2012). These findings suggest that activation of the GLP-1 receptor results in an elevated production of cAMP, activation of PKA and, subsequently, eNOS and the formation of NO. The latter can enhance endothelium relaxation through stimulation of soluble GC to increase cGMP in vascular smooth muscle cells (Liu et al., 2015). In addition to PKA activation, AMPK and Akt may also be involved in this process. Exenatide is found to stimulate proliferation of human coronary artery endothelial cells through PKA-PI3K/Akt-eNOS activation pathways (Erdogdu et al., 2010), and GLP-1 receptor agonists are able to stimulate eNOS activation and NO production through AMPK phosphorylation in humans (Krasner et al., 2014; Koska et al., 2015). This may be the direct result of AMPK on eNOS, or an indirect effect by activation of uncoupling protein-2 and subsequently reducing the amount of reactive oxygen species and production of vasoconstrictive mediators (Liu et al., 2015; Tang et al., 2016). In contrast, there is also evidence that NO is not involved in the effects of GLP-1 on endothelium-dependent vasodilatation. While infusion of GLP-1 relaxes the isolated rat femoral artery and the specific GLP-1 receptor antagonist exendin (9–39) completely inhibits this, the inhibition of NO does not (Nystrom et al., 2005). In healthy overweight men, the acute infusion of exenatide increases capillary perfusion in the skin, independent of NO (Smits et al., 2015). These discrepancies in NO-dependency may be a result of differences in study design, species, and types of vasculature or peptide used in these experiments. It has been suggested that increased secretion of atrial natriuretic peptide or diuretic effects are also prominently involved (Ryan and Acosta, 2015).

Besides NO, H_2_S and CO also contribute to the vasodilator effect of GLP-1 (Figure 9). In one study, exenatide causes a dose-dependent relaxation in isolated rat thoracic aorta (Sélley et al., 2014). Comparing the effects of the gasotransmitters leading to vasodilatation, H_2_S had the most remarkable. The cAMP/cGMP-PKA/PKG pathway is identified as an important mediator of the effects of the gasotransmitters. Inhibition of ATP-sensitive voltage-gated calcium-activated large conductance potassium channels, KCNQ-type voltage gated potassium channels and, foremost, the Na/Ca-exchanger decreases the vasodilatation evoked by exenatide. Taken together, this study identified possible cascades of GLP-1 leading to vasodilation and emphasizes the fact that, besides NO, other gaseous transmitters are involved in this process. To the best of our knowledge, this finding has not yet been confirmed by other studies.

The other effects of GLP-1 have been suggested to be organ protective effects due to antioxidant and anti-inflammatory effects, which may also involve effects of NO. Treatment of streptozocin-induced diabetic rats with vildagliptin, inhibitor of DPP-4, which inactivates GLP-1, reduces plasma TNF-α concentration and decreases NO concentration in serum and pancreatic homogenates compared with untreated diabetic rats (Akarte et al., 2012). Linagliptin, another DPP-4 inhibitor, decreases superoxide dismutase levels and increases NOS levels in hemodialysis patients (Kimura et al., 2016).

## Glucagon-Like Peptide 2

GLP-2 is a 33 amino acid peptide which is also produced by L-cells. Only 5–10% of the nutrients reach the distal part of the small intestine unabsorbed. In humans, GLP-2 secretion increases in response to exposure to unabsorbed isocaloric meals rich in carbohydrate or fat. Protein rich meals are poor stimulants (Xiao et al., 1999; Thulesen, 2004). GLP-2 plasma levels decrease in response to a fasting period (Drucker and Yusta, 2014). Following nutrient-induced release, GLP-2 effects are mediated by the G-protein-coupled GLP-2 receptor (GLP-2R) which is expressed in the GI-tract to enteroendocrine cells, sub-epithelial myofibroblast cells, and in the neurons of the enteric nervous system (Guan et al., 2006). GLP-2 has various physiological effects, including stimulation of growth and repair of intestinal epithelium, maintenance of mucosal integrity, upregulation of nutrient absorption, slowing of gastric emptying, reduction of gastric secretions, as well as effects on blood flow and blood pressure, bone resorption inhibitory effects and suppression of food intake (Dubé and Brubaker, 2007; Janssen et al., 2013). Understanding the GLP-2 pathways and developing compounds to manipulate the downstream pathway accordingly can lead to new treatments, especially as pre-clinical models: post-operative ileus, GI mucositis and conditions of altered intestinal permeability. The problem however is the short half-life time of GLP-2 (Salaga et al., 2017).

**FIGURE 10:**
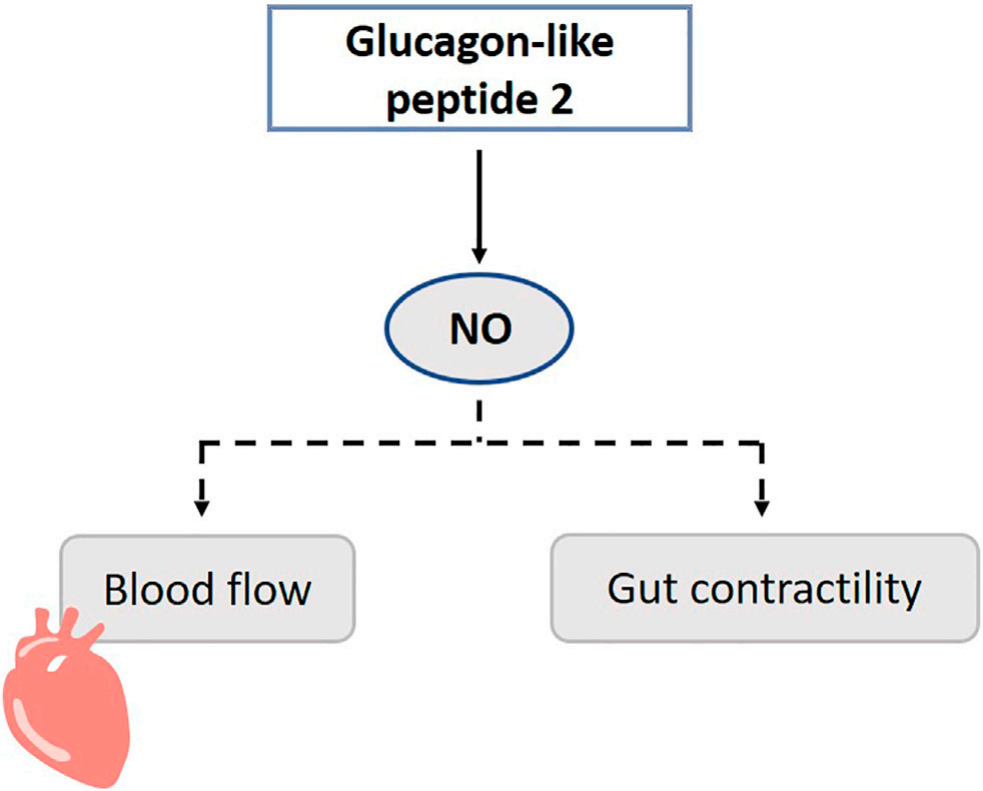
Role of gasotransmitters on glucagon-like peptide 2’s (GLP-2) physiological functions. Dotted lines represent first evidence for the presence of the pathway. GLP-2 promotes NO release, and this may increase blood flow and contractility of the gut. NO, Nitric oxide.

Infusion of GLP-2 acutely and dose-dependently increases intestinal blood flow, which is important for example to improve nutrient absorption. GLP-2Rs are co-localized with eNOS, nNOS and acetylcholine immune-responsive neurons in the myenteric and submucosal neurons of the small intestine. GLP-2 upregulates eNOS expression and stimulates NO release (Guan et al., 2006) (Figure 10). In the colon, activation of the GLP-2R rather antagonizes acetylcholine function and is NO-independent (Guan et al., 2003b; Amato et al., 2010). In rats, GLP-2 infusion increases blood flow in the superior mesenteric artery, but not in the inferior mesenteric or carotid artery. Only a higher dose of l-NAME can partly block the increased blood flow (Bremholm et al., 2009). It must be mentioned that rats are less sensitive for GLP-2 in comparison to mice. Besides inducing a local intestinal increase of blood flow, GLP-2 increases glucose uptake, eNOS mRNA and phosphorylation (eNOS-Ser1117) (Guan et al., 2003b; Guan et al., 2006). The effect of GLP-2 on contractility of the gut is also believed to act via the NO-mediated pathway in the myenteric plexus (Cinci et al., 2011).

In cisplatin treated mice, GLP-2 is able to counteract both the mucosal gastric fundus damage, and the neuropathy, respectively by preventing the epithelium thickness decrease and by protecting nNOS neurons (Pini et al., 2016). This data suggests that GLP-2 plays a role in maintenance in myenteric neurons including nNOS neurons. GLP-2 enhances the expression of iNOS through stimulating the activity of TGFβ-Smad2/3 signaling in osteoclasts *in vitro*, which may contribute to the inhibition of the proliferation of osteoclasts and which may provide potential therapeutic targets for the treatment of osteoporosis (Lu et al., 2018).

Although GLP-2 is shown to promote NO release, the primary relevance of the NO-dependent signaling of GLP-2 seems to be restricted (Cinci et al., 2011; Moloudi et al., 2011). It is hypothesized that this is also a permissive factor for other functions of GLP-2 such as intestinal growth (Dubé and Brubaker, 2007). Besides NO, several other downstream mediators of GLP-2 are known, including IGF-1 and -2, keratinocyte growth factor, and VIP (Rowland and Brubaker, 2011). It should be considered that the Ser1177 is also a phosphorylation site for Akt, and Akt is a downstream kinase of e.g. the IGF-1 receptor.

## Summary

Gut peptides are important regulators for several physiological processes ranging from muscles contractions or relaxations, systemic or local blood flow, anti-inflammatory actions, feeding behavior, glucose metabolism and many more. In the case of aberrant peptide production, the system becomes disturbed and a therapy needs to be started. The development of drugs targeting peptide receptors is a challenging process. Gaseous neurotransmitters are important for intracellular signaling, and increasing evidence emerges that these are involved in gut peptide release and their physiological functions. These small molecules may be easier to target for pharmacological intervention once their pathways to alter gut peptide signaling are better understood. A major challenge will be the widespread influence of gasotransmitters, in -and outside the gastrointestinal tract, which may thus be associated with loss of organ or process selectivity.
